# Characteristics of Heat Resistant Aluminum Alloy Composite Core Conductor Used in overhead Power Transmission Lines

**DOI:** 10.3390/ma13071592

**Published:** 2020-03-31

**Authors:** Kun Qiao, Anping Zhu, Baoming Wang, Chengrui Di, Junwei Yu, Bo Zhu

**Affiliations:** 1 School of Mechanical, Electrical & Information Engineering, Shandong University, Weihai 264209, China; 2 Carbon Fiber Engineering Research Center, Shandong University, Jinan 250061, China; 3 School of Materials Science and Engineering, Shandong University, Jinan 250061, China

**Keywords:** aluminum alloys, composite materials, epoxy resins, power cables, transmission lines

## Abstract

The heat resistant aluminum alloy wire composite material core conductor (ACCC/HW) which was used in overhead transmission lines is developed and studied in this work. The composite material core is carbon fiber/glass cloth reinforced modified epoxy resin composite. Tensile stress tests and stress-strain tests of both composite core and conductor are taken at 25 °C and 160 °C. Sag test, creep test and current carrying capacity test of composite conductor are taken. The stress of composite conductor are 425.2 MPa and 366.9 MPa at 25 °C and 160 °C, respectively. The sag of conductor of 50 m length are 95 mm, 367 mm, and 371 mm at 25 °C, 110 °C, and 160 °C, respectively. The creep strain are 271 mm/km, 522 mm/km, and 867 mm/km after 10 years under the tension of 15% RTS (Rated Tensile Strength), 25% RTS and 35% RTS at 25 °C, and 628 mm/km under 25% RTS at 160 °C, according to the test result and calculation. The carrying capacity of composite conductor is basically equivalent to ACSR (Aluminum Conductor Steel Reinforced). ACCC/HW is suitable in overhead transmission lines, and it has been used in 50 kV power grid, according to the results.

## 1. Introduction

In recent years, the electrical energy demand has significantly increased around the world [1]. Only in China, the total annual electricity consumption has grown from 4.2 × 10^12^ kWh to 6.3 × 10^12^ kWh between 2010 and 2017. However, the existing overhead power grids reached the transmission limit. There are two solutions to this problem, which are building new transmission lines or adopting large-capacity high-temperature low-sag (HTLS) conductors. At the same time, the most commonly used conductor ACSR, which has been studied and applied for more than one-hundred years [2,3,4,5,6], cannot meet the requirement. Accordingly, when considering land resources and economic benefits, it is necessary to develop new-style overhead conductors, which will increase the transmission capacity and not change the current power transmission distribution.

Several new kinds of conductor with composite materials have been developed to solve the above problem, such as ACCR (Aluminum Conductor ceramic fiber reinforced aluminum matrix Composite core Reinforced) [7,8] and ACCC (Aluminum Conductor Composite Core) [9,10,11,12]. Table 1 lists the structures and basic mechanical properties of ACSR, ACCR, and ACCC.

These conductors are composed of inner supporting cores and outer wires. The difference of the materials that were used in support cores is the key distinction between ACSR, ACCR, and ACCC. By comparison, ACCC with higher specific strength, lighter density, and higher tensile strength is more suitable for the HTLS overhead conductor. Because carbon fiber reinforced composite materials have the characteristics of light weight, high strength, corrosion resistance, and mass production. Additionally, carbon fiber reinforced composite materials are used in the fields of aerospace, sporting goods, rail transportation, pressure vessels, and so on [13,14,15].

ACCC reportedly exhibits high-strength, low-sag, large current carrying capacity, and high temperature resistance [11,12], which is composed of carbon/glass hybrid fiber reinforced epoxy resin composite and T-type soft aluminum wires, abbreviated to ACCC/TW (trapezoidal) [10]. The properties of ACCC/TW and its composite core have been studied for several years, such as mechanical properties [10], galvanic corrosion properties [11], excessive bending effect [12], and fatigue failure mechanism [17]. When compared to circular cross section heat resistant aluminum wires, the wires of this ACCC/TW conductor are made up of a softer aluminum alloy, and their trapezoidal shapes allows for them to be twisted more tightly. However, their stranding process is more difficult and more expensive. 

The cable composed of composite core and heat resistant aluminum alloy wires, abbreviated to ACCC/HW, might have better mechanical properties, heat resistance, and large current carrying capacity when compared with ACSR, because ACSR employs steel core and aluminum alloy wires, with the similar size, ACCC/HW can take more aluminum alloy wires. However, the performance of ACCC/HW has not been publicly reported.

The characteristics of ACCC/HW are tested and studied in this paper, such as tensile strength, strain-stress behavior, sag, creep deformation, and current carrying capacity at room temperature or at high temperature. At the same time, this work compares the properties of ACCC/HW and ACSR.

## 2. Materials and Methods 

### 2.1. Structure and Material

**Figure 1:**
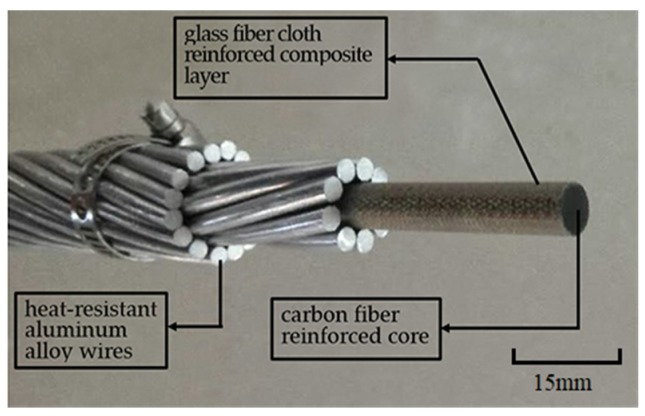
Conductor configuration of heat resistant aluminum alloy wire composite material core conductor (ACCC/HW).

**Figure 2:**
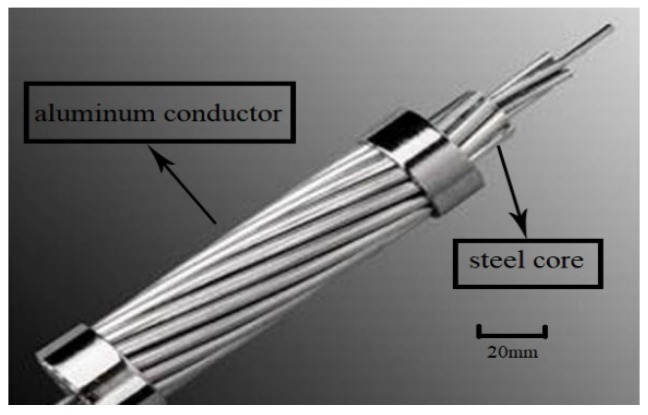
Conductor configuration of ACSR.

Figure 1 shows the conductor configuration of ACCC/HW in this work, and Figure 2 shows ACSR. ACCC/HW is composed of carbon fiber/glass fiber cloth reinforced modified epoxy resin composite core and two external layers of heat-resistant aluminum alloy wires. The difference between ACCC/HW and ACSR is the supporting core material. Table 2 lists the structural parameters of ACCC/HW and ACSR of the similar specification.

**Figure 3:**
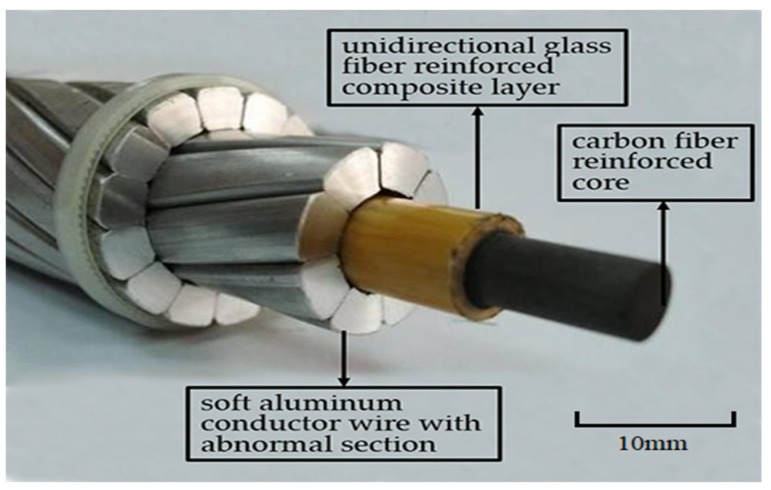
Conductor configuration of carbon/glass hybrid fiber reinforced epoxy resin composite and T-type soft aluminum wires (ACCC/TW).

Presently, the publicly reported ACCC overhead conductor is ACCC/TW, as shown in Figure 3. When compared with ACCC/TW, there are two features of ACCC/HW. Firstly, the conductive wire is heat-resistant aluminum alloy wire with circular section, and the wire used in ACCC/TW is soft aluminum wire with non-circular section. Secondly, the outer layer material of carbon fiber composite core used in ACCC/HW is bi-directional glass fiber cloth reinforced composite, and the outer material of ACCC/TW core is the unidirectional glass fiber reinforced composite.

The aluminum wire with non-circular section in ACCC/TW has certain radian, so the outer aluminum wires of ACCC/TW are stranded more closely. When comparing ACCC/HW with the same diameter, ACCC/TW contains more conductive aluminum area and it can conduct more electric quantity. However, the manufacturing process of soft aluminum wire with radian and non-circular section are more difficult, and the cost is relatively higher, especially in the case of ACCC/TW with small cross section. Therefore, with comprehensive consideration, ACCC/HW might be more suitable for the ACCC used in conductors of small size.

In this paper, the reinforcements of composite core are unidirectional carbon fiber and outer bi-directional glass fiber cloth. When compared with unidirectional reinforcements, the outermost bi-directional glass fabric can realize hoop enhancement and improve the anti-splitting performance and the anti-friction performance of the composite core. 

The pultrusion process prepared the carbon fiber/glass fiber cloth reinforced epoxy composite core used in ACCC/HW. The carbon fiber is 12 K commercial type manufactured. The glass fiber cloth is woven by non-alkali high-strength glass fiber. The epoxy resin system is composed of modified multi-functional group epoxy resin, modified anhydride curing agent, and other assistants. The aluminum wire is a conventional heat-resistant aluminum alloy wire. The strength of aluminum wire after stranding is 166 MPa (internal layer) and 171 MPa (outer layer), respectively, and the direct current resistivity at 20 °C is 0.028147 Ω·mm^2^/m.

### 2.2. Characterization

The ACCC/HW studied in this paper is only used in China now, so the test methods in this work are according to Chinese standard GB/T32502-2016 [18] “Overhead electrical stranded conductors composite core supported/reinforced”. Additionally, Figure 1 shows the ACCC/HW studied in this work is based on the physical model.

The basic mechanical property tests of both the composite core and ACCC/HW were taken in this study. The basic mechanical properties concluded tensile strength at 25 °C and 160 °C, and tensile-strain behavior at 25 °C. Deformation resistant property analysis and current-carrying capacity test of ACCC/HW were also taken. The deformation resistant properties included the sag at heating up temperature and the creep performances at 25 °C and 160 °C.

#### 2.2.1. Tensile Stress Test

**Figure 4:**
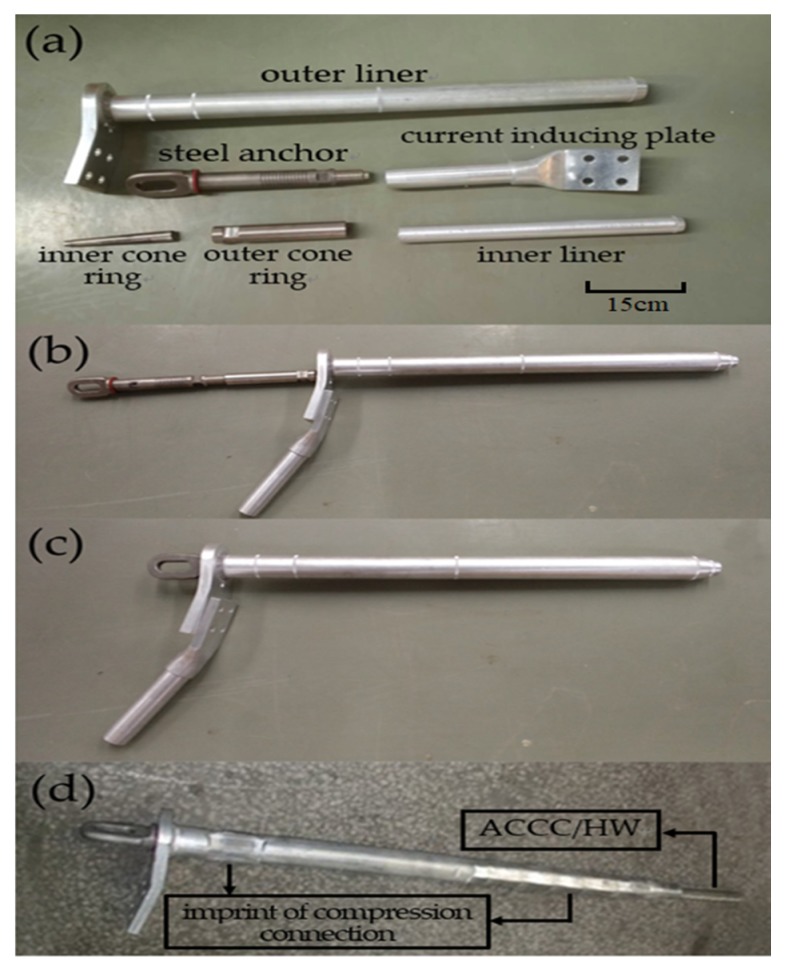
Special fittings used in this work. (**a**) detail sketch; (**b**) assembly process; (**c**) assembly process; (**d**) ready for use.

It is necessary to use special fittings, which should not only be closely connected with the composite core and the aluminum wires to effectively transfer load because of the difference between ACCC/HW and ACSR in structure, but also avoid the damage of carbon fiber composite core in the installation and use process. ACSR mainly adopts the compression connection mode, and the external pressure tightly combines the aluminum wire and the steel core because of the plastic deformation of metal materials. However, if ACCC/HW is connected in this way, the structure of the carbon fiber composite core will be destroyed, and the mechanical properties will be deteriorated. Therefore, special fittings were used, which were composed of inner cone ring, outer cone ring, steel anchor, inner liner, and outer liner, as well as current inducing plater, as shown in Figure 4. Inner cone ring is at the outside of the composite core, and outer cone ring is at the outside of inner cone ring, to protect composite core from destruction by extrusion. Inner liner and outer liner contact with heat-resistant aluminum alloy wires.

The tensile test and the stress-strain test were carried out on 500 kN tension test machine, and the strength was the average of three test results. In room temperature tensile test at 25 °C, the effective lengths of ACCC/HW and the composite core were 10 m and 1 m, respectively. In the high temperature tensile test at 160 °C, the effective lengths of ACCC/HW and the composite core were 12 m and 1 m, respectively. After ACCC/HW was heated to 160 °C through the 50 kV high current heating system and preserved for three hours, the tensile test of ACCC/HW was carried out. After the composite core heated to 160 °C through special heating furnace and preserved for 401 hours, the test of composite core was carried out.

#### 2.2.2. Stress-Strain Test

**Figure 5:**
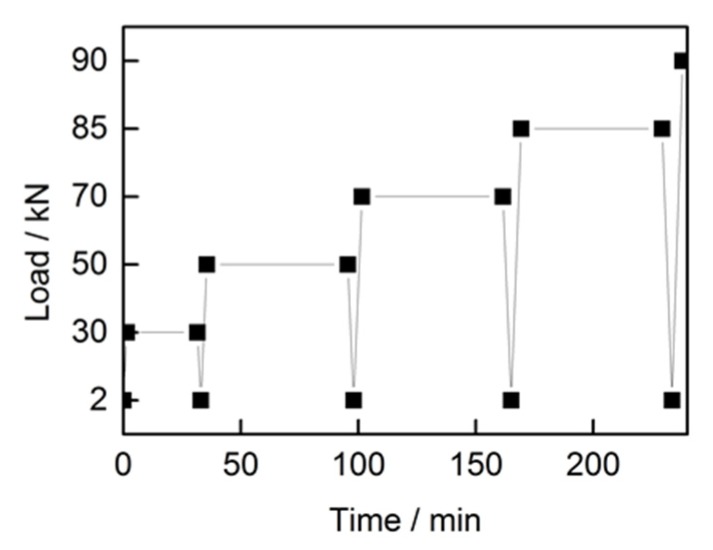
Stress-strain loading procedure of ACCC/HW.

ACCC/HW was applied load with four loading- load keeping-unloading cycles to characterize the stress-strain performance. The effective length of the ACCC/HW was 12 m, and the length of extensometer was 2 m. Firstly, 2% RTS (Rated Tensile Strength) was exerted to straighten the conductor, and the RTS is 100kN. Figure 5 shows the loading procedure. The stress-strain test of the composite core was loaded in only one cycle, and the effective length of the composite core was 5 m.

#### 2.2.3. Sag Test

In the sag test, ACCC/HW was heated through AC low voltage large current heating system. The temperature was measured through six pairs of thermocouples and the average temperature was taken. The test temperature was from 20 °C to 200 °C and the initial tension was 25% RTS. The sample test span was 50 m and the length of the heating span was 60 m. The sag of ACCC/HW at different temperatures was tested.

#### 2.2.4. Creep Test

The creep test of ACCC/HW was taken on the numerically controlled creep test machine according to IEC61395-1998 [19]. The effective length of ACCC/HW and the extensometer were 14m and 2000 ± 1 mm. The elongation of ACCC/HW under the tensions of 15% RTS, 25% RTS, and 35% RTS for different time were tested at 25 °C. The total test time was 1000 h. The high temperature creep test was carried out at 160 ± 3 °C and the tension was 25% RTS.

#### 2.2.5. Current-Carrying Capacity

The current-carrying capacity was tested according to IEC 1597-1995 [20]. Under the conditions of 25% RTS tension, the heating length of 13 m, no breeze, no sunshine, and natural convection, the current-carrying capacity of ACCC/HW was tested. Afterwards, the current-carrying capacity in different environments was calculated according to the calculation parameters that were recommended by China Electric Power Industry (wind speed was 0.5 m/s, sunshine intensity was 1000 W/m, conductor surface absorption coefficient was 0.9, conductor radiation coefficient was 0.9, ambient temperature was 20~45 °C, and conductor working temperature was 70~160 °C).

## 3. Results and Discussion

### 3.1. Basic Mechanical Property

**Figure 6:**
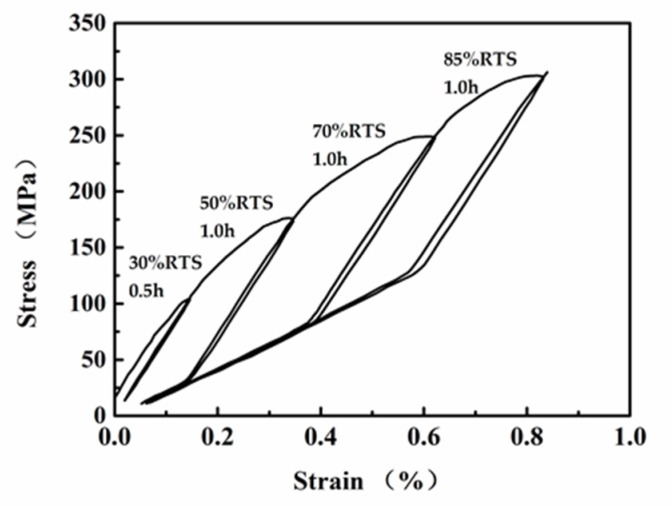
Stress-strain curve of ACCC/HW.

**Figure 7:**
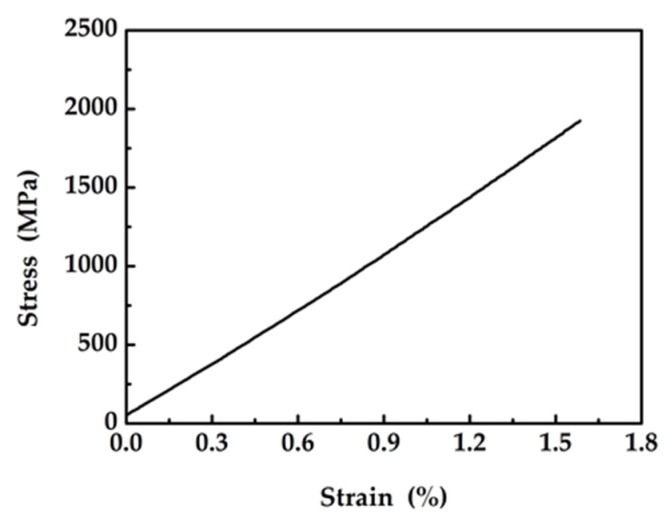
Stress-strain curve of composite core.

The fracture forces of ACCC/HW at 25 °C and at 160 °C are 121.9 kN (the average of 124.5 kN, 119.0 kN, and 122.1 kN) and 105.2 kN (the average of 105.9 kN, 104.6 kN, and 105.2 kN), and the stresses are 425.2 MPa and 366.9 MPa. The fracture forces of the composite core at 25 °C and at 160 °C are 113.5 kN (the average of 122.1 kN, 109.8 kN, and 108.8 kN) and 106.1 kN (the average of 106.1 kN, 104.6 kN, and 107.6 kN), and the stresses are 2597.3 MPa and 2427.9 MPa. Table 3 shows the stress-strain test results of ACCC/HW, and Figure 6 shows the curve. The elasticity modulus of the composite core is 137.7 GPa, and Figure 7 shows the stress-strain curve.

As shown in Figure 6, after loading to 30% RTS in the first cycle, the conductor is basically in the stage of elastic deformation. When the stress reaches 70 MPa, the slope of the stress-strain curve becomes smaller, but the change of slope is relatively small. During the second cyclic loading process to 50% RTS, the strain increases with the increase of stress at the beginning. Before the stress reaches 100MPa, the stress-strain loading curve basically coincided with the first unloading curve. After the stress reaches 100 MPa, the strain continuously increases with the increase of stress, but the increase speed obviously decreases. When the load reaches 50% RTS and the stress is 175 MPa, the load is no longer increased, and then kept for 60 min. In this case, the stress no longer increases, but the strain continuously increases. In the unloading process, the slope of the stress-strain curve is relatively large, and basically parallel to that before loading to 100 MPa in the second loading process. When the stress is unloaded to 30 MPa, the slope of the curve begins to decrease. During the third cyclic loading, the stress-strain curve changes for three times in the loading stage. At the initial stage of loading, the curve coincided with that in the last stage of the second unloading. When the stress reaches 3 0MPa, the slope of the curve suddenly increases and is then basically parallel to the curve in the initial stage of the second unloading process. When the stress reaches the peak point of the second curve at about 175 MPa, the slope of the curve decreases again, and the curve continuously increases slowly until the load reaches 70% RTS. Subsequently, the stress loading is stop, and the load is kept for 60 min. Similar to the second loading process, the stress no longer increases, while the strain continuously increases. The change of the curve in the third unloading process is basically the same as that in the second unloading process. At the beginning, the curve slope is large. After the stress reaches 83 MPa, the slope of the curve decreases until the stress is unloaded to the initial load. The curve change trend in the fourth process of loading and unloading is basically the same as that of the third time, with the difference in that the turning points of the loading curves are 83 MPa and 248 MPa, and the turning points of the unloading curves are 305 MPa and 129 MPa, respectively. The composite core is in an elastic deformation stage and the strain is below 1.1%, according to Figure 7.

Aluminum is metal material with elastic deformation and plastic deformation. Therefore, during the whole loading process of ACCC/HW, the aluminum strand has experienced two stages of elastic deformation and plastic deformation. In the first loading process, the curve changes according to the linear law, which indicated that the conductor is at the stage of elastic deformation. When the stress reaches 70 MPa, the curve is out of line, because plastic deformation occurs after the stress of the aluminum strand reaches the elastic limit. The 30% RTS is kept for 30 min. and then unloaded. It can be found that the unloading curve does not coincide with the loading curve, which results from the permanent plastic deformation of aluminum. The second loading process is basically the same as the first loading process. However, the elastic limit of aluminum is increased to 100 MPa because of the strain hardening of metal materials. In the process of unloading, the load of the aluminum strand is at the stage of plastic deformation, and the load is mainly supported by the composite core, so the slope of the unloading curve is larger. When the stress is unloaded to a certain stage, the aluminum strand is recovered to the elastic deformation stage and continues to bear load. The slope of the curve becomes smaller. Therefore, the inflection point occurred at 30 MPa of the stress-strain unloading curve. The third and fourth loading processes are basically the same as that of the second time.

The final modulus of ACCC/HW is only 71 GPa, which is slightly lower than the modulus of ACSR. However, Figure 6 obviously shows that the final non-recoverable plastic deformation of ACCC/HW is only 0.5‰, which is much smaller than the non-recoverable deformation of ACSR. Therefore, the safety of ACCC/HW is substantially improved.

### 3.2. Resistance to Deformation of ACCC/HW

**Figure 8:**
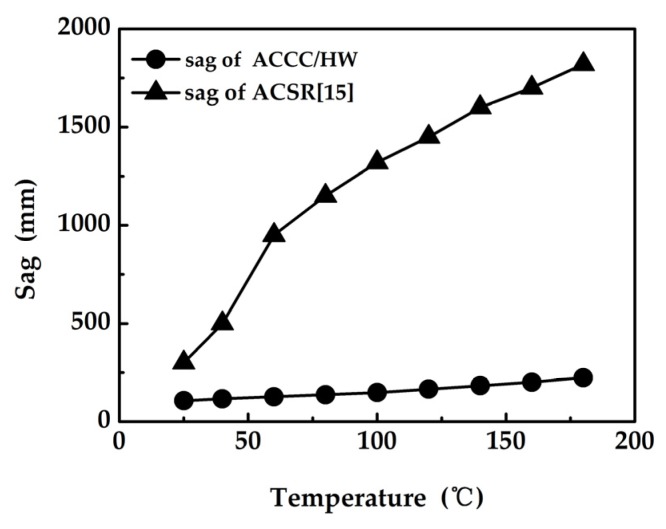
The sag curve of ACCC and ACSR with similar structure [21].

Figure 8 shows the sag test results of ACCC/HW and the sag curve of ACSR [21] with similar structure.

The sags of ACCC/HW at 25 °C, 110 °C, and 160 °C are 95 mm, 367 mm, and 371 mm, and the knee-point temperature is 110 °C, according to Figure 8. When the temperature is over 110 °C, the increase in sag is very small. The sags of ACSR with similar structure at 25 °C, 70 °C, 110 °C, and 160 °C are 231 mm, 1040 mm, 1420 mm and 1750 mm, respectively. The knee-point temperature of ACSR is 70 °C. Below the knee-point temperature, the sags of ACCC/HW and ACSR increases with the rise of temperature, and the sag of ACSR increases more rapidly. Above the knee-point temperature, the sag of the ACSR continuously increases, but the increase speed is smaller than that below the knee-point temperature. Additionally, above the knee-point temperature, the sag of ACCC/HW increases very slowly, which can be basically ignored. Based on the analysis, the sag of ACCC/HW is much smaller than that of ACSR, and it has higher safety.

The essential reason of sag is the thermal expansion property of material, and the thermal expansion is mainly determined by the thermal expansion coefficient of material. Below, the knee-point temperature point, the load is mainly borne by the core and aluminum conductor, and both the core material and aluminum conductor determine the sag. Above the transition temperature point, the load is mainly borne by the core, and the core material determines the sag. The thermal expansion coefficient of composite core, steel core, and aluminum conductor are 1.6 × 10^−6^, 11.6 × 10^−6^, and 23.6 × 10^−6^. Accordingly, the sag of ACCC/HW is smaller than that of ACSR below 110 °C, and much smaller than that of ACSR above 110 °C, only about 20% of that of ACSR. 

There are many ways to estimate the sag of ACSR [22], and several calculation formulas are given, such as (1) [22].

(1) $$D≅\frac{\omega S^{2}}{8H}$$

where D is the sag, ω is the resultant unit weight of the conductor, *S* is the span length, and H is the horizontal tension. In this sag test, the span length of ACCC/HW is 50 m, the total tension is 25 kN, and the weight is 742.9 kg/km. These test data do not match (1). Therefore, more sag test of the new type conductor should be taken to discover the law of sag phenomenon.

The sag characteristics of ACCC/HW that are discussed above are in short-term force. However, ACCC/HW in use is under a long-term tension state, and the deformation situation becomes very complicated. The deformation of the overhead conductor includes non-permanent deformation and permanent deformation. Non-permanent deformation includes elastic deformation and thermal deformation, the characterization physical quantities of which are the elasticity modulus E and the thermal expansion coefficient discussed above. Permanent deformation includes residual deformation and creep deformation [22]. The influence time of the residual deformation is very short when compared to the creep deformation. Therefore, the permanent deformation is mainly based on creep deformation in the long-term use.

When the temperature and tension are determined, the creep formula is formally consistent with the empirical creep formula of ACSR wire, as shown in (2) [23],

(2) $$\varepsilon =at^{\mu }$$

where, *ε* is the creep elongation in t hours, mm/km; *a* is the coefficient; *t* is the creep time, *h*; and, *μ* is the coefficient determined in the test.

The creep test results of the conductors are conducted with power fitting to derive the ACCC/HW creep equation. The creep equations at 25 °C and at 160 °C that are derived from the test results are (2) to (5).

(3) $$\varepsilon_{\left(10-10000\right)}=31.52t^{0.189}\left(15\%\;RTS\right)$$

(4) $$\varepsilon_{\left(10-10000\right)}=56.08t^{0.196}\left(25\%\;RTS\right)$$

(5) $$\varepsilon_{\left(10-10000\right)}=99.74t^{0.195}\left(35\%\;RTS\right)$$

(6) $$\varepsilon_{\left(10-10000\right)}=60.20t^{0.206}\left(25\%\;RTS,160\;{}^{{}^{\circ}}C\right)$$

The creep of the conductor is affected by the metallurgical creep and the strain due to the geometrical settlement [23]. The geometrical settlements of the conductors in the creep test are the same, so the material creep mainly causes the differences of test results. The creep of the conductor is the permanent deformation that is caused by slip and dislocation in the metal crystal of aluminum due to the long-time force. Even if the load is no longer growing in the test, the creep deformation will increase with time. However, the speed of creep deformation decreases with time. 

According to (3) to (6), the creep deformation of ACCC/HW increases at the power function with time. Coefficient a is the creep deformation of the conductor in one hour, and it is determined by the material, temperature, and stress. Exponent μ represents the growth rate of the creep deformation with time. It is affected by the material, structure, tension of ACCC/HW, as well as temperature. Coefficient a are 31.52, 56.08, and 99.74 at 15% RTS, 25% RTS, and 35% RTS, respectively. The initial creep deformation of the conductor increases with the increase of tension at 25 °C. Exponent μ are 0.189, 0.196, and 0.195 at 15% RTS, 25% RTS, and 35% RTS, respectively. The growth rate of creep deformation increases with tension growing below 25% RTS, and it remains largely unchanged between 25% RTS and 35% RTS. 

Under the tension of 25% RTS, the coefficient *a* at 25 °C and 160 °C are little different, which are 56.08 and 60.20. Exponent μ are 0.195 and 0.206, respectively. The creep speed of the conductor increases faster at 160 °C, but the difference from that at 25 °C is still quite small. This is because 160 °C is higher than the knee-point temperature, and the strength is mainly borne by the composite core.

According to the equations, it is derived that the creep deformation amounts of ACCC/HW under the tension of 15% RTS, 25% RTS, and 35% RTS in 10 years (87600 h) are 271 mm/km, 522 mm/km, and 867 mm/km. At 160 °C, the creep deformation under the tension of 25% RTS in 10 years is 628 mm/km, which is smaller than 0.6‰ of the length of ACCC/HW. The test result is much smaller than the creep deformation of aluminum or steel wires, because the creep of the composite core is very small. When the aluminum strand is deformed to a certain degree, the load is transferred to the carbon fiber composite core. In this case, the tensile strength of the aluminum strand is small and the creep is small, which leads to the small creep deformation of ACCC/HW.

### 3.3. Carrying Capacity

Carrying capacity is one of the main parameters for evaluating the power transmission capacity of conductors, and it is related to the conductor structure, surface state, resistance, operating temperature, environment temperature, sunshine intensity, and wind speed, etc. Table 4 lists the test results of the carrying capacity of ACCC/HW under the conditions of no wind, no sunshine, and natural convection, and Table 5 shows the calculation results.

Under the same conductor operating temperature, the carrying capacity decreases with the increase of the environment temperature, as shown in Table 5. At the same environment temperature, the carrying capacity increases with the increase of the conductor operating temperature. At the environment temperature of 40 °C, under the same calculation parameters, the calculated carrying capacity of 240/55 type ACSR with the similar structural parameters are 445A at 70 °C, 554A at 80 °C and 641A at 90 °C. Compared with the data in Table 5, under the conditions of operating temperature below 90 °C, the same environment temperature and the same operating temperature, the carrying capacity of ACSR is 1% greater than that of ACCC/HW. It can be considered that the carrying capacities of these two kinds of conductors are basically the same. The aluminum strands mainly determine the current carrying capacity. The sectional area of aluminum strands of ACCC/HW and ACSR are basically the same, so the current carrying capacities are also essentially the same. However, ACCC/HW does not have the magnetic hysteresis loss and thermal effect caused by the steel core.

In addition, a higher carrying capacity of the conductor can lead to serious heating situation and higher operating temperature. At a high temperature over 100 °C, the strength of ACSR reduces, the sag increases, and the security is greatly decreased. Moreover, the service life is also affected. Therefore, ACSR is not suitable for use in a high temperature environment. Many countries have raised the requirements for the maximum allowable operating temperature of ACSR [24]. The maximum allowable temperatures are 50 °C in Sweden, 70 °C in China, 80 °C in Germany, Holland and Switzerland, and 90 °C in the United States. The maximum allowable temperature limits the use of ACSR, as well as the carrying capacity of ACSR. In contrast, ACCC/HW is not subject to this limit. According to the test results, ACCC/HW has higher strength and smaller sag at 160 °C. Therefore, ACCC/HW is suitable for operating at high temperature. China has stipulated that the two long-term maximum allowable temperatures for ACCC are 120 °C and 160 °C. At the environment temperature of 40 °C, the carrying capacities of ACCC/HT are 816A at the operating temperature of 120 °C and 997A at the operating temperature of 160 °C. The high carrying capacity of ACCC/HW can be brought into play.

Therefore, there is little difference between ACSR and ACCC/HW in terms of carrying capacity at the temperature below 90 °C. However, the maximum allowable temperature of ACSR is 70 °C, while ACCC/HW can be used in a high temperature environment. ACCC/HW has greater carrying capacity at high temperature, which is of great significance in improving the transmission efficiency of the whole transmission grid.

## 4. Conclusions

ACCC/HW has been in a trial process in 50 kV line in China for more than 10 years. The stresses of ACCC/HW at 25 °C and 160 °C are 425.2 MPa and 366.9 MPa. The sag of ACCC/HW with length of 50 m are 95 mm at 25 °C, 367mm at 110 °C, and 371 mm at 160 °C, respectively. According to the formula that was obtained from the experimental results, the creep deformation amounts of ACCC/HW under the tension of 15% RTS, 25% RTS, and 35% RTS at 25 °C after 10 years are 271 mm/km, 522 mm/km, and 867 mm/km. The creep deformation under 25% RTS at 160 °C after 10 years is 628 mm/km. The carrying capacity of ACCC/HW is basically equivalent to traditional aluminum conductor steel reinforced (ACSR), and higher than ACSR at a high temperature. ACCC/HW shows excellent mechanical behavior and good current capacity, especially in high temperature operation. Therefore, ACCC/HW is suitable to use in the overhead conductor line, and it has great application potential. ACCC/HW behave the above good performance, because carbon fiber reinforced composite core is high-strength, low-sag, and high-heat resistant, and ACCC/HW takes advantage of carbon fiber reinforced composite. However, carbon fiber reinforced composite is more brittle when compared with metal, and more caution should be taken in ACCC/HW construction and maintenance, such as employing larger diameter reels and using special fittings. Moreover, more tests and studies of ACCC/HW should be taken to estimate the application performance. At the same time, future tests and studies should focus more on the overall toughness assessment and aging performances to evaluate and improve the safety and applicability.

## Figures and Tables

**Table 1:** Structures and basic mechanical properties of aluminum conductor steel reinforced (ACSR), (Aluminum Conductor ceramic fiber reinforced aluminum matrix Composite core Reinforced (ACCR), and Aluminum Conductor Composite Core (ACCC) [16].

Conductor Type	ACSR	ACCR	ACCC
Support core	Steel core	Ceramic fiber reinforced aluminum matrix core	Carbon fiber reinforced resin matrix core
Conductive wire	Aluminum	Aluminum alloy	Soft aluminum
Support core density (g/cm^3^)	7.8	3.3	1.8
Support core tensile stress (MPa)	1300	1275	2400
Support core modulus (GPa)	200	216	130

**Table 2:** Main structural parameters of ACCC/HW and ACSR of the similar specification.

Properties		ACSR(LION)	ACCC/HW
Aluminum wire	Number	30	25
	Diameter (mm)	3.18	3.68 (inner layer)
			3.43 (outer layer)
	Sectional area (mm^2^)	238.3	243.0
Supported core	Material of supported core	Steel	Carbon fiber reinforced composite
	Number	7	1
	Diameter (mm)	9.54	7.46
	Sectional area (mm^2^)	55.6	43.7
The conductor	Mass per unit length (kg/km)	1093.4	742.9
	Diameter (mm)	22.3	21.74
	Sectional area (mm^2^)	293.9	286.7

**Table 3:** Stress-Strain Test Results of ACCC/HW.

Tension and Preservation Time	Load (kN)	ACCC/HW	
		Stress (MPa)	Strain (%)
30%RTS 30 min	30	105.0	0.14
50%RTS 60 min	50	175.0	0.35
70%RTS 60 min	70	245.0	0.62
85%RTS 60 min	85	297.5	0.83
90%RTS 0 min	90	305.0	0.84

**Table 4:** Carrying capacity test results under the conditions of no wind, no sunshine, and natural convection.

Conductor Temperature/°C	Environment Temperature/°C	Carrying Capacity/A
61.0	23.0	329
80.5	23.0	468
100.1	23.1	565
120.8	23.4	631
140.2	23.8	698
160.7	23.9	767

**Table 5:** Carrying capacity results calculated according to the parameters that are recommended by China’s electric power industry.

Conductor Temperature (°C)	Environment Temperature (°C)					
	20	25	30	35	40	45
70	643	599	552	500	442	376
80	713	675	635	592	546	495
90	779	746	710	673	634	591
100	828	798	767	734	700	663
110	878	850	822	792	761	729
120	923	897	871	844	816	787
130	965	941	917	892	867	840
140	1004	982	960	937	913	889
150	1041	1021	1000	978	956	934
160	1077	1057	1038	1018	997	976
